# A Corpus-Based Comparison of the Pragmatic Use of *Qian* and *Hou* to Examine the Applicability of Space–Time Metaphor Hypothesis in Early Child Mandarin

**DOI:** 10.3389/fpsyg.2020.565763

**Published:** 2021-01-18

**Authors:** Linda Tsung, Dandan Wu

**Affiliations:** ^1^Faculty of Arts and Social Sciences, The University of Sydney, Darlington, NSW, Australia; ^2^School of Education, Macquarie University, Sydney, NSW, Australia

**Keywords:** spatial metaphor, temporal sequencing, temporal expression, early acquisition, corpus-based

## Abstract

The Universal Space–Time Mapping Hypothesis suggests that temporal expression is based on spatial metaphor for all human beings. This study examines its applicability in the Chinese language using the data elicited from the Early Childhood Mandarin Corpus (ECMC) (Li and Tse, 2011), which collected the utterances produced by 168 Mandarin-speaking preschoolers in a semistructured play context. The unique pair of Chinese words, *qian* (前/before/front) and *hou* (后/after/back), which can be used to express either time (before/after) or space (front/back) in daily communication, was the unit of analysis. The results indicated that: (1) there was a significant age effect in the production of “qian/hou,” indicating that the period before the age of 4.5 may be critical for the development of temporal and spatial expression; (2) the pair was produced to express time (before/after) much earlier than space (front/back), indicating that the expression of time might not necessarily be based on the spatial metaphor; and (3) the pair was used more frequently to express time (before/after) than space (front/back) by the preschoolers, thus challenging the hypothesis.

## Introduction

Space and time are the two fundamental and interrelated dimensions of human cognition and language production, with spatial terms being often used to describe the occurrence, sequence, and duration of events (Majid et al., 2013). This is because temporal relationships are abstract and invisible and thus have to be encoded into spatial terms using spatial metaphors, as suggested by the Conceptual Metaphor Theory (Lakoff and Johnson, 1980, 1999) and the Universal Space–Time Mapping Hypothesis (Fauconnier and Turner, 2008). Thus, it is widely accepted that temporal expression is based on spatial metaphor, and the concept of space is a precondition of temporal expression in all languages (Boroditsky, 2000). This theory has been confirmed by studies on the English language (Lakoff, 1994; Gentner et al., 2002; Zhang, 2003; Bender and Beller, 2014). However, recent studies have challenged this theory with evidence from other languages such as Amazonian (Sinha et al., 2011), Chinese (Chen, 2007), Japanese, and Marathi (Shinohara and Pardeshi, 2011). Chinese, featuring a pair of words—“*qian* 前” (before/front) and “*hou 后*” (after/back) that could be used to express both temporal (before/after) and spatial (front/back) concepts, provides a perfect case for empirically examining the applicability of this hypothesis (Yang and Xue, 2011; Tsung and Zhang, 2019). If the pair of words were used much earlier to express time rather than space, we could conclude that time expression might not necessarily be based on space metaphor. Accordingly, the premise of this theory would not be established; neither does the theory itself. Therefore, this study elicited the utterances with this pair of words from the Early Childhood Mandarin (Chinese) Corpus (ECMC) (Li and Tse, 2011) and analyzed their developmental patterns to test the hypothesis.

### The Space–Time Metaphor Hypothesis

Space and time are highly intercorrelated in human cognition and language; thus, their relationship has long been a philosophical inquiry topic, psychological exploration, and psycholinguistic study (Bottini and Casasanto, 2013). Lakoff and Johnson (1999) proposed the Space–Time Metaphor Theory to understand the asymmetric and sequential relationship between space and time and have empirical support from some metaphorical languages such as English. In English, the temporary expression is based on the spatial metaphor, using the words whose primary meaning is spatial—denotatively, developmentally, or historically (Clark, 1973). It is thus widely believed that the concept of space is acquired and expressed before that of time (Clark, 1973; Bowerman, 1996; Lan, 1999; Boroditsky, 2000). The space–time mappings and the asymmetry in the language (Lakoff and Johnson, 1999) have been verified with behavioral findings in psycholinguistics (Boroditsky, 2000), cognitive development (Casasanto et al., 2010), and psychophysics (Casasanto and Boroditsky, 2008; Bottini and Casasanto, 2010; Merritt et al., 2010). Bottini and Casasanto (2013) suggested that preschool and primary school children could ignore irrelevant temporal information when making judgments about space. Still, they might have difficulty ignoring spatial information when making judgments about time. This implies that the spatial system is acquired earlier than the temporal system.

This Space–Time Metaphor Hypothesis, however, has been challenged by many researchers with languages other than English. For example, Boroditsky (2001) compared Mandarin (the spoken form of Chinese) and English speakers’ conceptions of time and space. She found that English might prefer using the horizontal spatial metaphors to express time, for instance, “the good days ahead of us.” In contrast, the Chinese language tends to use vertical metaphors to express time, “the month above” means last month. Then, she concluded that English speakers conceived time differently from Mandarin speakers, indicating that language is a powerful tool in shaping habitual thoughts about abstract domains. However, this finding has been challenged by Chen (2007), who found that Chinese speakers used the horizontal spatial metaphors more often than the vertical metaphors and concluded that Chinese and English speakers shared the same way of thinking about time. Moreover, Kemmerer (2005) found that the temporal and spatial concepts could be represented and processed separately in the modern adult brain, thus challenging the Space–Time Metaphor Theory. However, these studies only tested adult subjects who had gone beyond the critical period of language acquisition. Although adults can process and express conceptions of time and space separately and independently, young children may not be able to do so. Therefore, we need to examine this theory using authentic data on young children.

### Matching Space–Time Concepts in *qian* (前) and *hou* (后) in Mandarin

Mandarin Chinese is the spoken form of Modern Standard Chinese (MSC) (Li, 2014), which provides an ideal arena for testing the cross-linguistic applicability of the Space–Time Metaphor Theory. MSC features three pairs of words that can express both time and space: *shang (上)- xia (下)*, *zuo (左)- you (右)*, *qian (前)- hou (后).* Among them, the pair of *qian (前)* and *hou (后)* has the strongest sense of space (Chen, 2007; Yang and Xue, 2011) thus has been widely used to express either the spatial contrast (FRONT/BACK) or the temporal sequencing (BEFORE/AFTER) (Gentner et al., 2002; Zhang, 2003). In particular, *qian* (前) signifies “before” in temporal sequencing and “in front of” in spatial sequencing, whereas *hou* (后) signifies “after” in temporal sequencing and “back” in spatial sequencing. Zhang (2003) has summarized the five types of temporal sequencing that could be expressed by the pair of *qian* (前) and *hou* (后) in MSC:

(1) Temporal adverbs:
(a) *yiqian* 以前 (before), *congqian* 从前 (before)
(b) *yihou* 以后 (after), *jinhou* 今后 (after)
(2) Temporal adjective prefixes:
(a) *qianren*前人 (predecessors), *qianqi*前妻 (ex-wife)
(b) *houji*后记 (postscript), *housheng*后生 (young man)
(3) Temporal postpositions:
(a) *wanfanqian*晚饭前 (before dinner)
(b) *wanfanhou晚饭后* (after dinner)
(4) Temporal prepositions:
(a) *qianbanye前半夜* (the first half of the night)
(b) *houbanye后半夜* (late night)
(5) Proverbs:
*kongqianjuehou空前绝后* (unprecedented)
*qianyinhouguo前因后果* (cause and effect)

In the above examples, the words *zhi qian* (之前), *zhi hou* (之后), *ran hou* (然后), and *zui hou* (最后) are used to convey relative temporal sequencing. In contrast, *qian mian* (前面) and *hou mian* (后面) can be used to express both spatial and temporal sequencing relations. Moreover, these temporal terms include words that refer to the future, such as *zhi hou* (之后), *ran hou* (然后), and *zui hou* (最后), and a set of words that refer to the past using the term *zhi qian* (之前). If the pair of words was widely used to express time much earlier than that of space in young children’s natural utterances, we would have to reject the premise of the Space–Time Metaphor Hypothesis—temporal expression based on space concepts. Therefore, the following research questions guided this study:

(1) Are there any age differences in Mandarin-speaking preschoolers’ production of temporal and spatial expressions using the pair of *qian* (前) and *hou* (后)? If yes, what are the developmental patterns?
(2) Do Mandarin-speaking preschoolers use the pair of *qian* (前) and *hou* (后) to express time earlier than that of space? If yes, what is the pattern of this preference?

In particular, we have the following hypotheses:

Hypothesis I: There will be a significant age effect in the pragmatic use of the target pair of words: *qian* (前) and *hou* (后).
Hypothesis II: The same words *qian* (前) and *hou* (后) will be used to express space much earlier than time.
Hypothesis III: The pair will be used more frequently to express space than time.

## Materials and Methods

### The Corpus

Li and Tse (2011) established the largest corpus on early child Mandarin, which includes 504 Chinese preschoolers aged from 2.5 to 5.5 years and randomly sampled from Beijing (*N*_*BJ*_ = 168), Hong Kong (*N*_*HK*_ = 168), and Singapore (*N*_*SG*_ = 168). Using Age (four groups), Gender (two), Society (three), and Language (Mandarin, Cantonese, and English) as the study variables, this corpus allows scholars to explore the age and gender differences in early psycholinguistic development and to conduct cross-linguistic and cross-society comparisons. So far, this corpus has generated six academic publications, exploring Chinese and English interrogative development in Beijing, Hong Kong, and Singapore preschoolers (Li et al., 2015, 2017, 2019), early acquisition of aspect markers and temporal adverbs in Mandarin- and Cantonese-speaking preschoolers (Tse et al., 2012; Liang et al., 2019), and early acquisition of Cantonese classifiers (Li and Wong, 2014). This study was based on the ECMC in Beijing (Li and Tse, 2011), which comprises 42 h of conversations between 168 Mandarin speakers aged from 2 to 5 years, with 21 boys and 21 girls in each age group. All participants were randomly sampled from eight preschools in Beijing, where Mandarin is the official and daily used language in China and the spoken form of Modern Standard Chinese (Li, 2014). All of the participants, their families, and the teachers spoke Mandarin.

### Communication Task

The participants were randomly paired (boy/girl, boy/boy, or girl/girl), and each pair was encouraged to play and talk with each other for 30 min in the same play context set up in the participants’ classroom. The context was furnished with toys, including cooking materials, food and fruits, furniture and electrical appliances, and hospital materials and vehicles. During playtime, their conversations were videotaped using a high-definition digital camera with two separate microphones and observed uninterruptedly by the researchers. The conversations were transcribed and checked by experienced research assistants (RAs). The spatial and temporal sequencing expressions were first identified by the RAs and then confirmed by a panel of Chinese linguists. Because the context was the same for every child, the children had equal chances of producing spatial and temporal expressions, thus ensuring an ideal setting for making comparisons.

### Coding System

The coding book was developed by the second author, verified by the first author, and reviewed by an independent psycholinguist. It was used to code all of the expressions collocated by the words *qian* (前) and *hou* (后) into four subtypes of time and one subtype of space: Time A for ran hou (然后), Time B for zui hou (最后), Time C for zhi hou (之后), and Time D for zhi qian (之前). Specifically, ran hou (然后) in Chinese also serve as a conjunction with the meaning of “and,” therefore, we excluded all the ran hou (然后) with conjunction meaning but only included the ran hou (然后) with the meaning of time. All of these terms are temporal sequencing expressions of future/past relations. A pair of space–term types for hou mian (后面) (Space A) and qian mian (前面) (Space B) was used to code expressions of spatial sequencing in terms of front/back relations (Table 1). All of the data were coded by the same RA to ensure 100% coherence in the coding based on the coding system.

**TABLE 1:** Inventory of temporal and spatial expression with qian/hou in the corpus (*N* = 168).

	Temporal Expression				Spatial Expression	
		
	Time A	Time B	Time C	Time D	Space A	Space B
Chinese	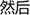	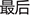	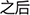	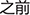	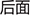	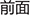
Pinyin	*ran hou*	*zui hou*	*zhi hou*	*zhi qian*	*hou mian*	*qian mian*
English	then	at last	after	before	back	in front of
Percentage	66.12	9.84	8.74	2.73	15.3	2.8

## Results

One hundred eighty-three cases of the use of the localizers *qian* (前) and *hou* (后) were elicited from the ECMC (Li and Tse, 2011). The pair of words was uttered by 72 Mandarin speakers across the four age groups (aged 2.5, 3.5, 4.5, 5.5), with 22 girls and 40 boys using the words correctly and 10 children misusing the terms. All of the usages were analyzed and placed within the typology shown in Table 1: (1) temporal expression: 然后 (then), 最后 (at last), 之后 (after), 之前 (before); and (2) spatial expression: 后面 (back) and 前面 (in front of). This section reports the results of the detailed statistical analyses.

Among the 183 cases of temporal and spatial expression, 66.12% was Time A, ran hou (然后), which means “then,” “afterward,” “after that,” “and then,” etc. The second most commonly used expression (15.3%) was Space A, hou mian (后面), which means “behind,” “at the back,” “in the rear,” “back,” etc. The third most commonly used expression (9.84%) was Time B, zui hou (最后), which means “at last,” “last,” “final,” “ultimate,” etc. The fourth most commonly used expression (8.74%) was Time C, zhi hou (之后), which means “later,” “after,” “afterward,” etc. The two least used terms, Time D “之前” (zhi qian, before) and Space B “前面” (qian mian, in front of), were related to the Chinese term “前.” The difference between the use of “前” and “后” may be associated with the participants’ cognitive level, which will be discussed in the next section.

### Age Differences in Temporal and Spatial Expression

As shown in Table 2, the number of participants who used the pair to express time varied across the age groups, with 10, 2, 20, and 29 cases from the age groups of 2.5, 3.5, 4.5, and 5.5, respectively. A set of chi-square tests was conducted to test the age differences, and a significant age effect was found for temporal expression [χ^2^ (3) = 3.79, *p* < 0.05] and Time A (*ran hou*) [χ^2^ (3) = 3.64, *p* < 0.05]. Non-significant differences were found for the other temporal and spatial expressions [χ^2^s (3) < 2.05, *p*s > 0.12]. In particular, about 69% of the 5.5 age group participants used temporal expressions, indicating that the 5–6-year-olds used this type of expression maturely and pragmatically. In contrast, only 4.8% of the participants in the 3.5 age group used temporal expressions. In addition, about 23.8% of the 5.5 age group participants used spatial expressions, indicating that most participants did not use the spatial expression. Similarly, only 7.1% of the participants in the 2.5 age group used spatial expressions. Furthermore, a jump from 11.9 to 23.8% was found in the spatial expression, which occurred between age groups 4.5 and 5.5, indicating that around age 5 may be a critical developmental period for spatial expression.

**TABLE 2:** Age differences in the temporal and spatial expressions with qian/hou.

		Aged 2.5		Aged 3.5		Aged 4.5		Aged 5.5		χ^2^
		(*N* = 42)		(*N* = 42)		(*N* = 42)		(*N* = 42)		
					
	In Chinese	*n*	%	*n*	%	*n*	%	*n*	%	
Time		10	23.8	2	4.8	20	47.6	29	69	3.79*
A	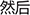	5	11.9	1	2.4	14	33.3	17	40.5	3.64*
B	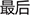	2	4.8	1	2.4	4	9.6	5	11.9	0.27
C/D	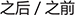	3	7.1	0	0	2	4.8	7	16.7	1.16
Space		3	7.1	4	9.6	5	11.9	10	23.8	1.53
*A*	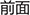	3	7.1	4	9.6	3	7.1	8	19	0.46
*B*	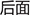	0	0	0	0	2	4.8	2	4.8	2.02

*p < 0.05; % = n/N.

In particular, in the 2.5 age group, 10 participants used temporal expressions: five for Time A (然后), two for Time B(最后), and three for Time C and Time D (之后/之前), whereas only three of this group used *qianmian* (前面) to express a spatial relationship, and no child used *houmian* (后面) in the communication. For example, one 2-year-old correctly used “*zhi qian* (之前)” in the utterance “*dan gao zai kao zhi qian*” (蛋糕在烤之前; English: before baking the cake) (Time D) to express the meaning of “before baking the cake.” In the 3.5 age group, two children used temporal expressions: one for Time A (然后) and one for Time B (最后), and no child used Time C/D (之后/之前). Only four participants used *qianmian* (前面) to express a spatial relationship, and no child used houmian (后面) in the communication. In the 4.5 age group, 20 children used temporal expressions: 14 for Time A (然后), four for Time B (最后), and two for Time C/D (之后/之前). Only five children used *qianmian* (前面) to express a spatial relationship, and two used *houmian* (后面) in communication. In the 5.5 age group, 29 children used temporal expressions: 17 for Time A (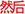), five for Time B (最后), and seven for Time C/D (之后/之前). Eight participants used *qianmian* (前面) (Space B) to express a spatial relationship, and two used *houmian* (后面) (Space A) in the communication.

Last, the analysis revealed two critical developmental periods. First, in the 4.5 age group, almost half (47.5%, 69%) of the children used temporal expressions, indicating that age 4 may be a critical developmental period for temporal expression development. Second, in the age groups before age 4.5, no child used a spatial expression with *houmian* (后面), indicating that age 4 may be a critical developmental period for this type of spatial expression. Therefore, this study’s results jointly indicated that age 4 might be a critical developmental period for temporal and spatial expressions. And Hypothesis I has been supported by the data of this study.

A 2 (gender) × 2 (expression) chi-square test was conducted to examine the gender differences in temporal versus spatial expression. The results indicated no significant gender difference, *χ*^2^(1) = 1.645, *p* = 0.147. For details, see Table 3.

**TABLE 3:** Gender differences in the temporal and spatial expressions with qian/hou.

English	Chinese	Boy (*N* = 84)		Girl (*N* = 84)	
			
		*n*	*%*	*n*	*%*
**Temporal word**		30	35.7	20	23.8
Time A	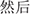	21	25	16	19
Time B	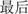	6	7.1	6	7.1
Time C/D	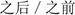	9	10.7	3	3.6
**Spatial word**		15	17.9	6	7.1
Space A	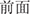	3	3.6	1	1.2
Space B	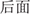	14	16.7	5	5.9

**%* = *n/N.**

### Preschoolers’ Pragmatic Preference for Temporal Expression

**FIGURE 1:**
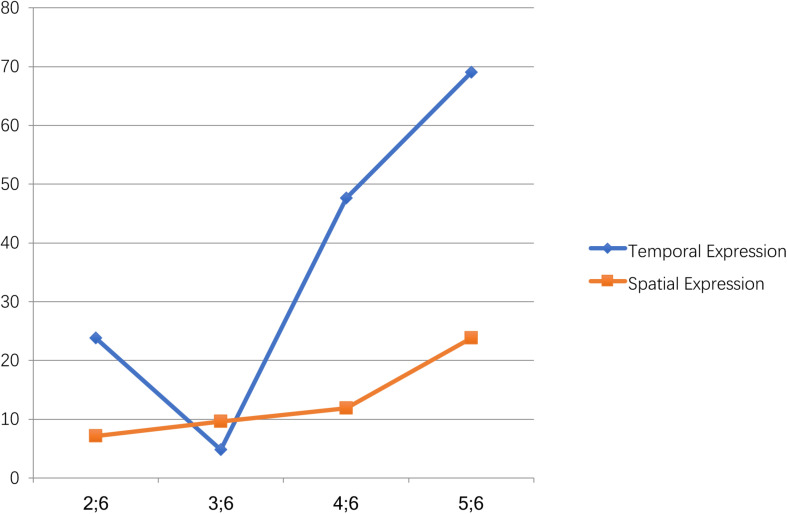
Developmental trends of temporal and spatial expression with qian/hou.

**FIGURE 2:**
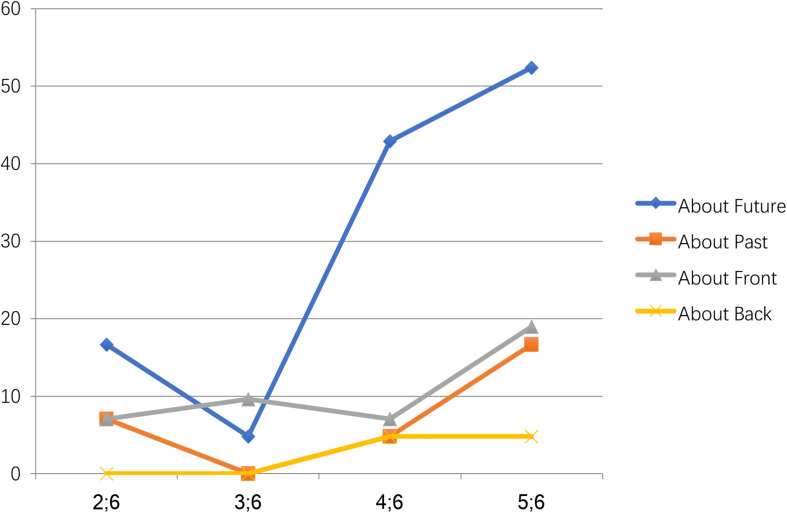
Age differences in the pragmatic functions of Qian/Hou.

Further analysis revealed that more temporal expressions were used within each age group than spatial expressions (Figure 1). The only exception was the 3.5 age group, which produced relatively more spatial than temporal expressions. In particular, in the 2.5 age group, seven children (16.7%) talked about the future, three (7.1%) talked about the past, three (7.1%) talked about the front, and none talked about the back. In the 3.5 age group, two children (4.8%) talked about the future, four (9.6%) talked about the front, and none talked about the past and the back. In the 4.5 age group, 18 children (42.9%) talked about the future, two (4.8%) talked about the past, three (7.1%) talked about the front, and two (4.8%) talked about the back. In the 5.5 age group, 22 children (52.4%) talked about the future, seven (16.7%) talked about the past, eight (19%) talked about the front, and two (4.8%) talked about the back. Significantly more Mandarin-speaking preschoolers preferred to talk about the future (time), and very few talked about the back (space) (Figure 2). This “future-preference” phenomenon might be linked to early cognitive development and warrants further study. All these findings indicated that Hypotheses II and III should be rejected in this study.

## Discussion

Time and space are two fundamental dimensions of human cognition and language, and their acquisition and expression have been a fascinating and important research topic. As the first comparison of temporal and spatial expressions using the same pair of words, *qian* (前) and *hou* (后), this study found significant age differences and remarkable developmental patterns in early child Mandarin. The findings did not support all the hypotheses (except for Hypothesis I). This section will discuss the major findings and their implications for future studies.

### The Developmental Pattern of Pragmatic Use

This study revealed a significant age effect in temporal expressions production, particularly Time A (*ran hou*). This finding suggested that the period between 2.5 and 5.5 might be critical for acquiring temporal expressions, especially future expressions, among Chinese preschoolers. However, no significant age differences were found in the production of spatial expressions, indicating that the period between 2.5 and 5.5 might not see any remarkable development in this regard. It was found that only a few children in the 5.5 age group (23.8%) were able to produce spatial expressions, indicating that the children in this age group were only beginning to develop their capacity to produce spatial expressions. Therefore, we could conclude that the Mandarin-speaking preschoolers began to produce temporal expression between the ages of 2.5 and 5.5, whereas they only began to produce spatial expression around age 5.5. This finding implies that Chinese children might acquire spatial expression capacity later than that for temporal expression. However, this finding needs to be further explored and verified with longitudinal studies.

### Pragmatic Preference for Temporary Expression

This study found that although the same pair of words could be used to express time and space, the participants preferred to express time (84.70%) more than space (15.30%). This finding implies that young Mandarin speakers might prefer to use the pair of Chinese words to express time, given that the research setting equally invited both temporal and spatial expressions. This finding could provide empirical evidence to support Boroditsky; Boroditsky’s (2000; 2001) hypothesis that “thinking about time does not necessarily require access to spatial schemas.” In addition, this finding has also provided alternatives to challenge the idea that spatial expression is the precondition or foundation for temporal expression (Wallentin et al., 2005; Kranjec et al., 2010). Nevertheless, it is also important to note that Boroditsky (2000, 2001) used an adult sample and experimental design, whereas this study used a sample of young children and a corpus design, which provided naturalistic and authentic data on early child language acquisition. In particular, this study found that even though children did not produce any spatial expressions using *qian* (前) and *hou* (后), they could use the related temporal expressions. This finding implies that temporal expression using the same words as a morpheme might not necessarily be an adaptive use of spatial metaphor.

This study has also provided new evidence to support Kemmerer’s (2005) hypothesis that the time and space domains might be represented and processed separately and independently in the brain. This separation in brain processing implies that temporal expressions’ processing may not necessarily depend on spatial expressions. If Kemmerer’s Hypothesis were true, Mandarin-speaking preschoolers would have developed their temporal and spatial expressions separately. Accordingly, the temporal expression of *qian* (前) and *hou* (后) would not be constrained by the spatial expression. Accordingly, it is natural and understandable that the Mandarin-speaking preschoolers in this study produced the temporal expression more and earlier than the spatial ones. However, because the corpus used in this study only included young children aged 2–5 years, leaving very younger children (0–2 years old) understudied. Thus, the possibility of the early production of spatial expression using the pair of qian/hou could not be ruled out, thus warranting future studies on this topic.

### Temporary Expression Produced Earlier Than Spatial Expression

This study found that the 2.5 age group produced considerably more temporal sequencing expressions than the spatial ones, indicating that young Mandarin speakers tended to produce temporal expressions more and earlier (Figures 1, 2). This production difference in the early years indicated that the temporal expression might have occurred earlier than the spatial expression of *qian/hou*. Accordingly, this finding has provided new evidence to support Boroditsky; Boroditsky’s (2000; 2001) statement that thinking about time does not necessarily require access to spatial schemas, as temporal language is not adapted from spatial sequencing. In addition, Boroditsky (2001) has attributed temporal expression preference in her experiments to the adults’ experiences as she believed that the concept was before the language. Experiences in different cultural and linguistic contexts will cause different conceptualization and expression of time and space. Chinese people’s view of time and space might be different from that of English speakers. Therefore, Boroditsky’s study on adults might not control the confounding effects of culture and sociolinguistic contexts. In contrast, this corpus-based study was designed to examine the language acquisition of young Mandarin speakers who had far less experience in using language than adults. Therefore, this study could provide authentic evidence on language acquisition during the early years, demonstrating the true relationship between temporal and spatial expression. According to this study, when the young children did not produce any spatial expressions using *qian/hou*, the related temporal expressions had been produced. This finding implies that temporal expressions using the same words as a morpheme might not be the adaptive use of spatial metaphor, thus challenging the Universal Space–Time Mapping Hypothesis.

## Conclusion, Limitations, and Implications

The pair of Chinese words qian/hou could be used to express either time or space, thus providing an ideal case to test the cross-linguistic applicability of the Space–Time Metaphor Hypothesis. First, this corpus-based study found a significant age effect in the pragmatic use of the target pair of words, indicating that the period before the age of 4.5 might be critical for developing temporal and spatial expression. Second, the pair was used to express time (before/after) much earlier than space (front/back), indicating that t might not necessarily be based on the spatial metaphor. Third, the pair was used more frequently to express time (before/after) than space (front/back) by the preschoolers, thus challenging the hypothesis.

This study has some limitations. First, as the corpus collected only a sample of the entire target language (rather than the whole), the sample size must be increased and data should be gathered from more typical everyday settings to gain a more representative sample. Second, the sample was cross-sectional rather than longitudinal, making the evidence less robust for understanding the long-term developmental trend. Third, it would be perfect if similar corpus data could be collected from adult participants; otherwise, we could not judge whether the pragmatic preference for temporal expression would be a norm in Mandarin-speaking. Last, the younger children (0–2 years old) should also be included in this study, as they might have also produced the spatial and temporal expressions using qian/hou words.

Nevertheless, as the first comparison of temporal and spatial expressions using the same pair of words, this study has initiated a new experimental paradigm for studying the complicated relationships among cognition, language, and pragmatics in the early years. This study might not provide sound evidence to overthrow the Space–Time Metaphor Hypothesis completely but has provided an exceptional case to challenge the universal applicability of this hypothesis. This study’s finding has at least indicated that the Space–Time Metaphor Hypothesis might not be applicable in early child Mandarin. Therefore, more cross-linguistic and cross-contextual studies are urgently needed.

## Data Availability Statement

The data analyzed in this study is subject to the following licenses/restrictions: the dataset is confidential. Requests to access these datasets should be directed to dandan.wu4@students.mq.edu.au.

## Ethics Statement

The studies involving human participants were reviewed and approved by Ethical Committee of the Faculty of Education in The University of Hong Kong. Written informed consent to participate in this study was provided by the participants’ legal guardian/next of kin.

## Author Contributions

LT and DW completed the whole manuscript writing. DW designed the study and analyzed the data by the guidance of the LT. Both authors contributed to the article and approved the submitted version.

## Conflict of Interest

The authors declare that the research was conducted in the absence of any commercial or financial relationships that could be construed as a potential conflict of interest.
